# Artificial neural network models for prediction of cardiovascular autonomic dysfunction in general Chinese population

**DOI:** 10.1186/1472-6947-13-80

**Published:** 2013-07-31

**Authors:** Juanmei Liu, Zi-Hui Tang, Fangfang Zeng, Zhongtao Li, Linuo Zhou

**Affiliations:** 1 Department of Endocrinology and Metabolism, Fudan University Huashan Hospital, Shanghai 200040, China; 2 Department of Computer Science, Youzhou Vocational and Technology Collage, Yongzhou City, Hunan 425000, China

**Keywords:** Cardiovascular autonomic dysfunction, Artificial neural network, Prediction model, Chinese population

## Abstract

**Background:**

The present study aimed to develop an artificial neural network (ANN) based prediction model for cardiovascular autonomic (CA) dysfunction in the general population.

**Methods:**

We analyzed a previous dataset based on a population sample consisted of 2,092 individuals aged 30–80 years. The prediction models were derived from an exploratory set using ANN analysis. Performances of these prediction models were evaluated in the validation set.

**Results:**

Univariate analysis indicated that 14 risk factors showed statistically significant association with CA dysfunction (P < 0.05). The mean area under the receiver-operating curve was 0.762 (95% CI 0.732–0.793) for prediction model developed using ANN analysis. The mean sensitivity, specificity, positive and negative predictive values were similar in the prediction models was 0.751, 0.665, 0.330 and 0.924, respectively. All HL statistics were less than 15.0.

**Conclusion:**

ANN is an effective tool for developing prediction models with high value for predicting CA dysfunction among the general population.

## Background

The prevalence of cardiovascular autonomic (CA) dysfunction is increasing worldwide, particularly in developing countries. The disease is not only a major factor in the cardiovascular complications of diabetes mellitus (DM) [1], but it also affects many other major segments of the general population, such as the elderly and patients with hypertension (PH), metabolic syndrome (MetS), and connective tissue disorders [2-4]. CA dysfunction has become a major health concern in China following rapid changes in lifestyle. The prevalence of CA dysfunction in diabetic patients was found to be 30–60% [1]. CA function testing using HRV is sensitive, noninvasive, and reproducible; therefore, it is easily applicable for screening a large number of individuals in the general population [5].

In clinical medicine, a prediction model refers to the type of medical research study using which researchers try to identify the best combination of medical signs, symptoms, and other findings that may be used to predict the probability of a specific disease or outcome [6]. These models may aid the clinician in the decision-making process regarding clinical admission, early prevention, early clinical diagnosis, and application of clinical therapies. An artificial neural network (ANN) refers to a mathematical model inspired by biological neural networks [7]. ANNs employ nonlinear mathematical models to mimic the human brain’s own problem-solving process, by using previously solved examples to build a system of “neurons” that makes new decisions, classifications, and forecasts [8]. According to learning paradigms, each corresponding to a particular abstract learning task, these are supervised learning, unsupervised learning and reinforcement learning. ANN is often applied to model complex relationships between inputs and outputs or to find patterns in data. In clinical medicine, ANN models have been applied in the diagnosis of diseases such as myocardial infarction [9]. ANN models have also been successfully used to predict trauma mortality and in clinical decision-making in the management of traumatic brain injury patients [10,11]. A previous study developed ANN models to be used in the prediction of living setting after hip fracture [12]. However, no studies in literature have used ANN for modeling of CA dysfunction prevalence in the general population. The aim of this study was to develop a prediction model for CA dysfunction using ANN analysis.

## Methods

### Study population

The study protocol was approved by the Ethics Committee of Huashan Hospital, Shanghai, China. We analyzed a previously constructed database of a CA dysfunction survey carried out in a random sample of middle-aged Chinese individuals. Participants were recruited from three communities in Shanghai, China, primarily from the Baoshan District area. Participants with undiagnosed CA dysfunction, aged 30–80 years, were included in this study. A total of 3,012 subjects were invited to a screening visit between 2011 and 2012. Subjects with potential confounding factors that may influence cardiac autonomic function were excluded from the study. A total of 2,092 (69.46%) participants with complete baseline data were obtained. Written consent forms were obtained from all the patients before the start of the study.

The subjects were interviewed to document their medical histories and medication, history of smoking habits, laboratory assessment of cardiovascular disease risk factors, and standardized examination for HRV. All study subjects underwent a complete CAF evaluation after fasting for eight hours. The evaluation included: (a) history and physical examination, (b) heart rate and blood pressure, (c) fasting serum glucose and insulin, and (d) fasting plasma lipids. The body mass index was calculated as the weight in kilograms divided by the square of the height in meters. Fasting plasma glucose (FPG) was quantified by the glucose oxidase procedure, and HbA1c was measured by ion-exchange high-performance liquid chromatography (HPLC; Bio-Rad, Hercules, CA, USA). The serum total cholesterol (TC), high-density lipoprotein (HDL) cholesterol, triglyceride (TG) levels, creatinine (Cr), and uric acid (UA) levels were measured enzymatically with a chemical analyzer (Hitachi 7600–020, Tokyo, Japan). Systolic and diastolic blood pressure (BP) values were the means of two measurements obtained by the physician on the left arm of the seated participant. The day-to-day and inter-assay coefficients of variation at the central laboratory in our hospital for all analyses were between 1% and 3%.

Short-term HRV test was applied to evaluate CA function. HRV was measured non-invasively by power spectral analysis. Subjects were studied while awake and in the supine position after 20 minutes of rest. Testing times were from 8:00 AM to 11:00 AM, and 1:30 PM to 4:30 PM. A type-I FDP-1 HRV BRS non-invasive detection system was used (version 2.0; Department of Biomedical Engineering, Fudan University, Shanghai, China). Electrocardiography and respiratory signals and beat-to-beat blood pressure were continually and simultaneously recorded for 15 minutes by using an electrosphygmograph transducer (HMX-3C) placed on the radial artery of the dominant arm and an instrument respiration sensor. Short-term HRV analysis was performed for all the subjects using a computer-aided examination and evaluation system for spectral analysis to investigate changes in autonomic regulation.

### Definition

PH was defined as blood pressure ≥140/90 mmHg or history of anti-hypertensive medication. BMI was classified on the basis of Chinese criteria: normal, <24.0 kg/m^2^; overweight, ≥24.0 kg/m^2^ <28.0 kg/m^2^; obese, BMI ≤ 28.0 kg/m^2^. Fasting plasma glucose (FPG) levels ≥ 5.6 mmol/L were considered high. Central obesity was defined using ethnicity-specific values: waist circumference (WC) ≥90 cm in men or ≥80 cm in women [13]. Serum triglyceride (TG) levels ≥1.7 mmol/L were considered high. Serum high-density lipoprotein-cholesterol (HDL-C) levels <0.9 mmol/L in men or <1.0 mmol/ L in women were considered low. Diabetes was diagnosed by the oral glucose tolerance test (OGTT) and determined by either HbAlc ≥ 6.5% or the use of insulin or hypoglycemic medications. Individuals meeting three or more of the updated National Cholesterol Education Program/Adult Treatment Panel III criteria (WHO Western Pacific Region obesity criteria) were diagnosed as having MetS [13]. CAN was diagnosed on the basis of at least two abnormal cardiovascular autonomic reflex test results [1].

### Statistical analysis

The Kolmogorov-Smirnov test was used to determine whether continuous variables followed a normal distribution. Variables that were not normally distributed were log-transformed to approximate normal distribution for analysis. The results are expressed as means ± standard deviation or medians, unless otherwise stated. The subject characteristics according to MetS severity scores were assessed using one-way analysis of variance (ANOVA) for continuous variables and the *χ*^2^ test for categorical variables. Potential CA dysfunction risk factors, which are known clinically and in literature to be associated with CA dysfunction, were selected for the evaluation. These factors included age, gender, BMI, WC, current smokers (yes/no), resting HR, diabetes, hypertension, blood glucose profile, lipid profile, and renal profile. Univariate analyses were performed to estimate the significant predictors of CA dysfunction.

#### Artificial neural network models

A computerized random number generator was used to select three-fourths of the patients to make up the exploratory set to develop prediction models. The remaining one-fourth of the patients comprised the validation set. The exploratory and validation sets were similar for all developed models.

**Figure 1:**
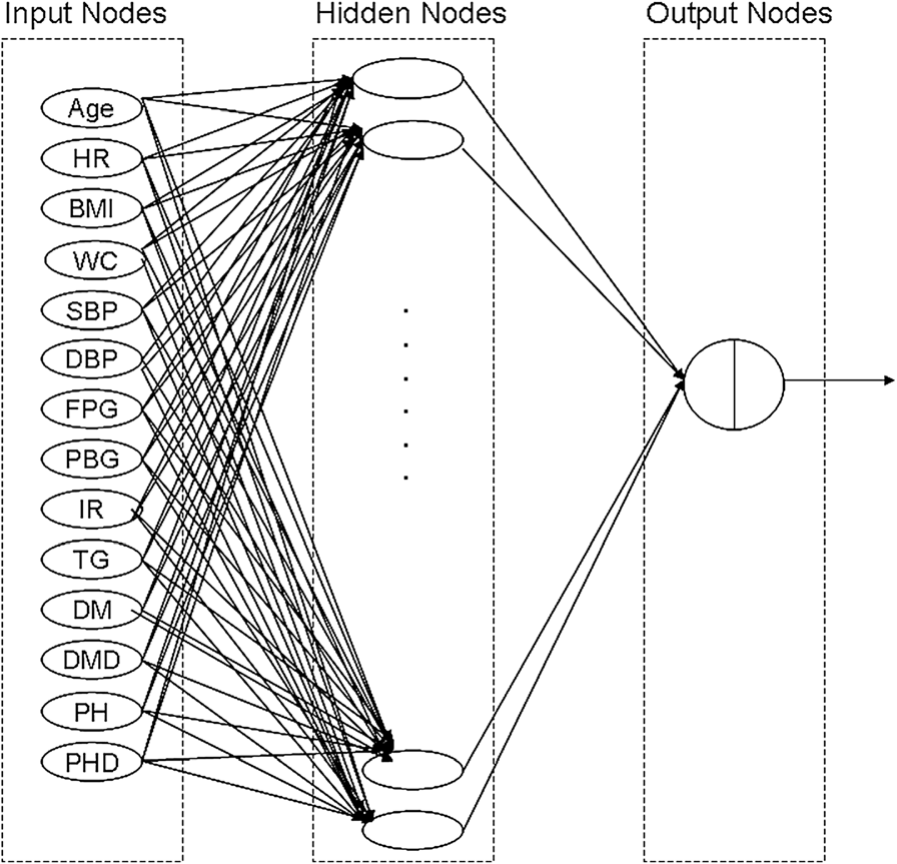
**Artificial network model showing input variables (nodes), hidden nodes, and connection weights with output node for data on CA dysfunction.** The ANN model including 14 input nodes, 18 hidden nodes and 1 output node. BMI- Body mass index, WC-waist circumference, SBP- systolic blood pressure, DBP- diastolic blood pressure, FPG- fasting plasma glucose, PBG- plasma blood glucose, IR-insulin resistance, TG- triglyceride, UA- uric acid, HR-heart rate, PH- Hypertension, DM- Diabetes, PHD- Hypertension duration, DMD- Diabetes duration.

The ANN applied in this study was a standard feed-forward, back-propagation neural network with three layers consisted of an input layer, a hidden layer, and an output layer. The input layer contained 14 input neurons, the hidden layer contained 18 neurons, and the output layer contained 1 output neuron (Figure 1). The number of hidden layer neurons was determined through trial and error, since no accepted theory currently exists for predetermining the optimal number of hidden layer neurons [14]. The number of hidden layer neurons was selected to lead to a predictive network with the best sensitivity and specificity. During the training, the learning rate and momentum for network training were set to 0.20 and 0.9, respectively. To obtain the connection weights, the network first underwent a training process using the back-propagation of error method, which employs the generalized delta learning rule. This is an iterative process by which input derivation sets are used to the ANN, and outputs are calculated. The output is then compared to the desired output, and the connection weights are adjusted based on the error in output. A validation dataset was developed to avoid an over-fitting ANN model. In general, one-fourth of the patients were randomly selected from the exploratory set. The training was run until a minimum average square error (MSE) of <0.001 or an increasing MSE was found in the validation dataset.

A developed prediction model generated the probability value for CA dysfunction from output node. The probability value was calculated for each participant. The performance of the prediction model developed in this study was evaluated in the validation set.

#### Model evaluation

Discrimination and calibration were both measured. Discrimination refers to the ability of a model to distinguish between individuals with and without CA dysfunction. The discriminatory power of the models was analyzed using a receiver-operating characteristic (ROC) curve and area under the curve (AUC). ROC curves were constructed by plotting true positives versus the false positive fraction. Sensitivity (the probability of a positive test given the individual has the disease), specificity (the probability of a negative test given the individual does not have the disease), positive predictive value (the probability of having the disease given a positive test), and the negative predictive value (the probability of not having the disease given a negative test) were calculated for each cutoff score. The cutoff score that gave the maximum sum of sensitivity and specificity was considered optimum [15]. Calibration refers to how accurately the models predicted over the entire range. The calibration of models was computed using the Hosmer-Lemeshow (HL) test, which is a single summary measure of the calibration and is based on comparing the observed and estimated prevalence of disease grouped by estimated prevalence [16]. The HL statistic follows a χ^2^ distribution, with degrees of freedom equal to two less than the number of groups. The overall accuracy (ratio of summary of the number of true positive and true negative results to the total sample size) of the prediction model was calculated by comparing the predicted values with the actual events.

All parameters of discrimination were evaluated in the five validation sets. The mean the AUC, sensitivity, specificity, and predictive values were calculated and compared using paired *t* tests (P < 0.05). Odds ratios (OR) with 95% confidence intervals (CI) were calculated for the relative risk of predictors with outcome. Results were analyzed using the Statistical Package for Social Sciences for Windows version 16.0 (SPSS; Chicago, IL, USA). The BP ANN models were developed using Matlab 7.0.

## Results

Table 1 indicated that baseline clinical characteristics of the 2092 subjects. The entire sample included 705 men and 1387 women (mean age, 60.42 ± 8.68 years; Table 1). A total of 387 (18.51%) individuals had CA dysfunction. The mean FPG, TC, and TG levels were 5.53, 5.32, and 1.71 mmol/L in total sample, respectively. The HRV components decreased with age (data not shown). The HR of individuals with CA dysfunction was very significantly higher than that of individuals without CA dysfunction (P < 0.001). Most HRV parameters were lower in individuals with CA dysfunction than in those without CA dysfunction (P <0.01 for all).The prevalence of PH, DM, and MetS in the entire sample was 46.65, 21.33, and 39.82%, respectively. The baseline characteristics were similar between the exploratory and validation sets (p < 0.05; data not shown).

**Table 1:** Subject characteristics

**Variables**	**Entire sample**	**Individuals with**	**Individuals**	***P***
		**CA dysfunction**	**without CA**	value*
			**dysfunction**	
N	2092	387	1705	
Age	60.42 ± 8.68	62.94 ± 8.43	59.85 ± 8.64	<0.001
Gender male,%	705 (33.7%)	143 (36.95%)	562 (32.96%)	0.134
Height cm	161.46 ± 7.79	161.45 ± 7.83	161.46 ± 7.78	0.987
Weight kg	63.26 ± 10.61	64.85 ± 11.09	62.9 ± 10.47	0.001
BMI kg/m^2^	24.21 ± 3.36	24.84 ± 3.69	24.07 ± 3.26	<0.001
WC cm	85.07 ± 9.70	87.68 ± 9.93	84.48 ± 9.54	<0.001
SBP mmHg	127.62 ± 18.68	132.95 ± 20.02	126.41 ± 18.14	<0.001
DBP mmHg	79.83 ± 9.69	81.28 ± 9.93	79.50 ± 9.61	0.001
Laboratory assays				
FPG mmol/L	5.53 ± 1.81	6.12 ± 2.53	5.4 ± 1.57	<0.001
PBG mmol/L	7.67 ± 3.56	9.03 ± 4.53	7.36 ± 3.22	<0.001
FINS IU/L	7.19 ± 11.82	9.17 ± 21.66	6.74 ± 8.01	<0.001
TC mmol/L	5.32 ± 1	5.39 ± 1.05	5.31 ± 0.98	0.142
TG mmol/L	1.71 ± 0.98	1.9 ± 1.17	1.67 ± 0.92	<0.001
HDL mmol/L	1.36 ± 0.32	1.34 ± 0.32	1.36 ± 0.33	0.203
LDL mmol/L	3.19 ± 0.77	3.23 ± 0.8	3.18 ± 0.76	0.229
SCr μmol/L	77.81 ± 26.04	78.51 ± 21.93	77.65 ± 26.89	0.561
Ccr	82.01 ± 30.84	81.31 ± 32.65	82.17 ± 30.42	0.624
UA μmol/L	281.21 ± 83.79	285.97 ± 86.04	280.13 ± 83.25	0.216
HRV measurement				
HR beats/min	72.42 ± 10.13	79.7 ± 11.26	70.77 ± 9.08	<0.001
TP ms^2^	873.95 ± 702.47	315.87 ± 410.75	1000.63 ± 693.2	<0.001
LF ms^2^	190.98 ± 207.88	43.97 ± 57.29	224.34 ± 215.08	<0.001
LF nu	21.33 ± 10.66	15.97 ± 9.19	22.54 ± 10.6	<0.001
HF ms^2^	183.05 ± 219.43	41.82 ± 59.63	215.11 ± 229.61	<0.001
HF nu	20.67 ± 13.25	17.06 ± 13.98	21.49 ± 12.94	<0.001
LF/HF	1.7 ± 1.98	2.37 ± 3.32	1.55 ± 1.48	<0.001
Medical history				
Smoking yes,%	306 (14.63%)	62 (16.02%)	244 (14.31%)	0.390
PH yes,%	976 (46.65%)	241 (62.27%)	735 (43.11%)	<0.001
DM yes,%	446 (21.33%)	139 (35.92%)	307 (18.02%)	<0.001
MetS yes,%	833 (39.82%)	204 (52.71%)	629 (36.89%)	<0.001

Note: * present the difference between individuals with and without cardiovascular autonomic (CA) dysfunction.

*BMI:* Body mass index, *WC:* waist circumference, *SBP:* systolic blood pressure, *DBP:* diastolic blood pressure, *FPG:* fasting plasma glucose, *PBG:* plasma blood glucose, *FINS:* fasting blood insulin, *IR:* insulin resistance, *TC:* serum total cholesterol, *TG:* triglyceride, *UA:* uric acid, *HDL:* high-density lipoprotein cholesterol, *LDL:* low density lipoprotein cholesterol, *SCr:* serum creatinine, *Ccr:* creatinine clearance rate, *HR:* heart rate, *TP:* total power of variance, *LF:* low frequency, *HF:* high frequency, *MetS:* metabolic syndrome, *PH:* Hypertension, *DM:* Diabetes.

To estimate the potential risk factors of CA dysfunction, univariate analysis was performed in the entire sample. These potential risk factors contained the demographic parameters, blood glucose, and insulin function parameters; lipid profiles; and medical history factors. The result indicated that 14 potential risk factors—age, HR, BMI, WC, SBP, DBP, FPG, PBG, IR, TG, DM and its duration, and PH and its duration—were significantly associated with CA dysfunction (P < 0.05 for all parameters; Table 2).

**Table 2:** Univariate analysis for CA dysfunction

**Variables**	***β***	***P *value**	**OR (95% CI)**
Age	0.428	<0.001	1.53 (1.35–1.75)
HR	0.859	<0.001	2.36 (2.09–2.67)
BMI	0.273	0.001	1.31 (1.13–1.53)
WC	0.510	<0.001	1.67 (1.3–2.14)
SBP	0.018	<0.001	1.02 (1.01–1.02)
DBP	0.019	0.001	1.02 (1.01–1.03)
FPG	0.450	<0.001	1.57 (1.39–1.78)
PBG	0.475	<0.001	1.61 (1.41–1.83)
IR	0.279	<0.001	1.32 (1.20–1.46)
TG	0.336	0.003	1.40 (1.12–1.75)
DM	0.936	<0.001	2.55 (2.00–3.25)
DM duration	0.412	<0.001	1.51 (1.30–1.76)
PH	0.779	<0.001	2.18 (1.74–2.73)
PH duration	0.356	<0.001	1.43 (1.28–1.59)

Note: *HR:* heart rate, *BMI:* body mass index, *WC:* waist circumference, *SBP:* systolic blood pressure, *DBP:* diastolic blood pressure, *FPG:* fasting plasma glucose, *PBG:* plasma blood glucose, *IR:* insulin resistance, *TG:* triglyceride, *PH:* Hypertension, *DM:* Diabetes, *IR:* insulin resistance.

For developing a prediction model, five exploratory sets were generated using a computerized random calculator. Each exploratory set consisted of more than 1500 individuals. A total of 15 individuals with 14 risk factors developed from univariate analysis had missing data, so that 2077 individuals were available to form the dataset for development of the artificial neural network prediction model. The same exploratory and validation sets were applied for the artificial neural network model and a total of five ANN models were developed. Every trained ANN included 14 input nodes, 18 layer nodes, and 1 output node (Figure 1). For training ANN, 101–112 echoes were performed and the MSE ranged from 0.12–0.13. Five validation sets were developed, all of which consisted of more than 500 subjects. The area under ROC curve ranged from 0.738–0.789 (Table 3). At the respective optimal cutoff points, when applied to the validation sets, the sensitivity and specificity of the ANN models were 67.7–82.1% and 64.7–70.4%, respectively. The positive and negative predictive values ranged from 30.1–37.3% and 89.8–94.0%, respectively.

**Table 3:** Prediction models using artificial neural network

	**Model 1**	**Model 2**	**Model 3**	**Model 4**	**Model 5**	**Mean ± SD**	**95% CI**
**AUC**	0.738	0.763	0.737	0.783	0.789	0.762 ± 0.025	0.732–0.793
**Cutoff**	0.234	0.229	0.216	0.227	0.175	0.216 ± 0.024	0.187–0.246
**Sensitivity**	0.694	0.789	0.677	0.777	0.821	0.751 ± 0.065	0.667–0.828
**Specificity**	0.694	0.663	0.647	0.704	0.618	0.665 ± 0.035	0.622–0.709
**Yuden Index**	0.388	0.452	0.324	0.481	0.439	0.413 ± 0.063	0.334–0.491
**PPV**	0.332	0.339	0.301	0.373	0.321	0.330 ± 0.026	0.298–0.361
**NPV**	0.912	0.935	0.898	0.932	0.94	0.924 ± 0.018	0.902–0.945
**HL Statistics**	14.64	8.143	8.421	7.424	7.196	9.165 ± 3.103	5.313–13.017
**Accuracy**	0.695	0.685	0.651	0.714	0.661	0.681 ± 0.026	0.650–0.713

Note: *AUC:* area under the receiver-operating curve, *PPV:* positive predictive value; *NPV:* negative predictive value.

The diagnostic accuracies of the ANN models are compared in Table 3. The mean AUC was 0.762 for ANN models (Table 3). The mean optimal cutoff points for ANN models were 0.216. The mean sensitivity and specificity of the ANN models were 75.1% and 66.7%, respectively. The mean PPV and NPV were 0.330 and 0.924, respectively. The HL statistics of the prediction model using ANN analysis were <15.0, indicating that these prediction models showed good fit. The mean values of accuracy were 0.681 for prediction models developed using ANN approaches.

## Discussion

We conducted a study to develop the prediction models using ANN analyses based on a dataset obtained from a large-scale population-based cross-sectional study. The database consisted of 2,092 participants from the Chinese population. The participants were a good representative sample across the country, and the prediction model developed in this study might work well even outside the studied areas in China. The prediction model was developed in the exploratory set and the performance of the developed model was evaluated in the validation set.

The important finding of this study was that the prediction models developed using ANN analyses have high value in predicting CA dysfunction in the general population. The mean AUCs were 0.762 for ANN models. In general, a prediction model has a high value for predicting outcomes if AUC was more than 0.70 for this model. The mean sensitivity of the models was >75%. Additionally, the mean specificity of the two models was > 65%. These models were good-fit models based on the large-scale dataset (HL statistics < 15.0). The mean accuracy of predictive model was near 0.70. However, these prediction model had not very high predictive value (AUC >0.90). this is partly because genetics risk factor was not considered [17]. CA dysfunction was a human complex disease attributed to genetics and environmental factors or/and its interactions. Missing genetics data was a limitation of this study. Anyway, these findings support that ANN models have high predictive value and can be applied to clinical decision making. These findings support evidence that ANN models were applied to clinical predictive practice.

Currently, LR and ANN are the most widely used models in biomedicine [11,16,18]. LR can generate excellent models and can serve as a commonly accepted statistical tool. Its popularity may be attributed to the interpretability of model parameters and its ease of use. However, the LR model uses linear combinations of variables, so it is not adept at modeling grossly nonlinear complex interactions [8]. ANNs are flexible nonlinear systems, and therefore they may be better suited than LR-based models to predict outcomes when the relationships between the variables are complex, multidimensional, and nonlinear, such as those encountered in complex biological systems [7]. The advantages and disadvantages of ANN models can be classified according to the following criteria [19]. First, development of an ANN model would require less domain knowledge. ANNs are ideally suited to modeling complex or unclear relationships since no prior knowledge of the underlying data is required. ANNs therefore can model any implicit interactions among input variables commonly encountered in medical data. In general, ANNS was prone to over-fitting model. Development of an ANN model requires more computation time. ANN models are commonly called black boxes.

ANN models have its advantages, and the selection of a model should be based on these advantages and the intended purpose of the study. ANNs would be particularly useful when there are implicit interactions and complex relationships in the data. In clinical practice, ANN models may be used complementarily to aid in decision making. ANN models have the potential to help physicians with respect to understanding CA dysfunction risk factors and diagnosis. These findings should be reproducible in other populations. This and similar models may emerge to be of considerable practical value in patient triage. Suitable ANN software should be designed for clinical practice. However, building an ANN or another hybrid technique that incorporates the best features of both the LR and ANN models might result in the development of the ideal prediction model for CA dysfunction.

This study has several limitations. First, the dataset was based on a cross-sectional study and could have been biased by selection. Furthermore, the temporal sequence between risk factors and outcome was questionable. Second, participants were recruited from Shanghai and external validation was not performed. Therefore, further investigation is required to determine the generalizability of our prediction model. Third, the association between HbAlc was not analyzed in the present study, because data on HbAlc levels were unavailable. Finally, it is important to mention that our study was performed on the Chinese population, and our findings may not be relevant to people of other ethnicities.

## Conclusion

In conclusion, we developed ANN models for the prediction of CA dysfunction in the general population by using a cross-sectional dataset. The performance of the ANN model with high value predicted CA dysfunction. Validation of the models’ prediction performance in an external validation set will be conducted. A larger and more complete database may be used to further clarify ANN models in terms of prediction of the clinical outcome following CA dysfunction.

## Abbreviations

ANN: Artificial neural network; AUC: Area under the receiver-operating curve; BP: Blood pressure; BMI: Body mass index; BSA: Body surface area; CA: Cardiovascular autonomic; Ccr: Creatinine clearance rate; CI: Confidence intervals; Cr: Creatinine; DM: Diabetes; FPG: Fasting plasma glucose; HbAlc: Glycosylated hemoglobin; HDL: High-density lipoprotein cholesterol; HF: High frequency; HL: Hosmer-lemeshow; HOMA-IR: Homeostasis model assessment insulin resistance estimate; HRV: Heart rate variability; IDF: International diabetes federation; LDL: Low-density lipoprotein cholesterol; LF: Low frequency; MetS: Metabolic syndrome; MLR: Multivariable logistic linear regression; OGTT: Oral glucose tolerance test; OR: Odds ratios; PBG: Postprandial blood glucose; PH: Hypertension; RACE: Rapid autonomic cardiovascular evaluation; TC: Serum total cholesterol; TG: Triglyceride; WC: Waist circumference; UA: Uric acid.

## Competing interests

The authors declare that they no competing interests.

## Authors’ contributions

Z-HT, JL, FZ and ZL carried out the molecular genetic studies, participated in the sequence alignment and drafted the manuscript. LZ, Z-HT participated in the design of the study and performed the statistical analysis. LZ, JL conceived of the study, and participated in its design and coordination and helped to draft the manuscript. All authors read and approved the final manuscript.

## Pre-publication history

The pre-publication history for this paper can be accessed here:

http://www.biomedcentral.com/1472-6947/13/80/prepub
